# DNA Methylation Profiling across the Spectrum of HPV-Associated Anal Squamous Neoplasia

**DOI:** 10.1371/journal.pone.0050533

**Published:** 2012-11-30

**Authors:** Jonathan M. Hernandez, Erin M. Siegel, Bridget Riggs, Steven Eschrich, Abul Elahi, Xiaotao Qu, Abidemi Ajidahun, Anders Berglund, Domenico Coppola, William M. Grady, Anna R. Giuliano, David Shibata

**Affiliations:** 1 Department of Gastrointestinal Oncology, H. Lee Moffitt Cancer Center and Research Institute, Tampa, Florida, United States of America; 2 Department of Cancer Epidemiology, H. Lee Moffitt Cancer Center and Research Institute, Tampa, Florida, United States of America; 3 Department of Biomedical Informatics, H. Lee Moffitt Cancer Center and Research Institute, Tampa, Florida, United States of America; 4 Department of Anatomic Pathology, H. Lee Moffitt Cancer Center and Research Institute, Tampa, Florida, United States of America; 5 Division of Gastroenterology, University of Washington, Seattle, Washington, United States of America; 6 Clinical Research Division, Fred Hutchinson Cancer Research Center, Seattle, Washington, United States of America; South Texas Veterans Health Care System and University Health Science Center San Antonio, United States of America

## Abstract

**Background:**

Changes in host tumor genome DNA methylation patterns are among the molecular alterations associated with HPV-related carcinogenesis. However, there is little known about the epigenetic changes associated specifically with the development of anal squamous cell cancer (SCC). We sought to characterize broad methylation profiles across the spectrum of anal squamous neoplasia.

**Methodology/Principal Findings:**

Twenty-nine formalin-fixed paraffin embedded samples from 24 patients were evaluated and included adjacent histologically normal anal mucosa (NM; n = 3), SCC-*in situ* (SCC-IS; n = 11) and invasive SCC (n = 15). Thirteen women and 11 men with a median age of 44 years (range 26–81) were included in the study. Using the *SFP_10_ LiPA HPV-typing system*, HPV was detected in at least one tissue from all patients with 93% (27/29) being positive for high-risk HPV types and 14 (93%) of 15 invasive SCC tissues testing positive for HPV 16. Bisulfite-modified DNA was interrogated for methylation at 1,505 CpG loci representing 807 genes using the Illumina GoldenGate Methylation Array. When comparing the progression from normal anal mucosa and SCC-IS to invasive SCC, 22 CpG loci representing 20 genes demonstrated significant differential methylation (p<0.01). The majority of differentially methylated gene targets occurred at or close to specific chromosomal locations such as previously described HPV methylation “hotspots” and viral integration sites.

**Conclusions:**

We have identified a panel of differentially methlylated CpG loci across the spectrum of HPV-associated squamous neoplasia of the anus. To our knowledge, this is the first reported application of large-scale high throughput methylation analysis for the study of anal neoplasia. Our findings support further investigations into the role of host-genome methylation in HPV-associated anal carcinogenesis with implications towards enhanced diagnosis and screening strategies.

## Introduction

Anal squamous cell cancers (SCC) account for 4% of all lower gastrointestinal tract malignancies in the United States with an estimated 6,230 new cases and 780 deaths expected in 2012 [1]. The incidence of anal cancer continues to rise steadily, with an average increase of 2.4% per year reported between 1992 and 2009 [2], [3]. Infection with human papillomavirus (HPV) has been demonstrated to be the primary causative agent in the development of SCC of the anogenital tract, including cancers of the cervix, vulva, vagina and anus [4]. Although infection with HPV is a common event, the subsequent development of cancer is rare, suggesting that additional molecular events are required for malignant transformation [5].

Anal intraepithelial neoplasia (AIN) is the precursor lesion of invasive anal SCC. It is generally thought that high grade AIN (HGAIN) or carcinoma *in situ,* but not low grade lesions, are at risk of malignant progression to invasive anal SCC. It is estimated that the risk of malignant progression of HGAIN is approximately 10% but may be higher in immunocompromised individuals [6]. Consequently, the optimal method for screening and managing patients with HGAIN (e.g. prophylactic treatment vs. observation) remains somewhat controversial [7], [8]. Molecular biomarkers of anal neoplasia have the potential to more accurately risk stratify HGAINs and also may improve the low sensitivity of HGAIN diagnosis found with anal PAP smears, thus reducing the need for costly invasive biopsies [9].

It is well recognized that genetic mutations occur in cancer cells and that these exert disease-associated changes in gene expression and/or function. Very little is known about the specific genetic events that drive anal carcinogenesis; although it has been reported that alterations in *TP53, DCC, APC* and *FHIT* may be contributing factors [10], [11]. Cancer cells also exhibit aberrant epigenetic alterations which appear to play a prominent role in cancer development. DNA methylation is a key aberrant epigenetic event that has been documented in virtually every tumor type studied and is amongst the earliest disease-associated changes observed during tumorigenesis [12]. HPV may influence the host transcriptome via a number of epigenetic mechanisms [5], [13] including HPV E7 oncoprotein-mediated alterations in the activity of DNA methyltransferases (DNMTs) [14], [15], histone deacetylases (HDACs), and pCAF acetyltransferase [14], [16].

Despite the likely importance of aberrant DNA methylation in the pathogenesis of anal SCC, there has only been one report investigating methylation in anal cancer. Zhang et al. evaluated the methylation status of 11 candidate genes identified from studies of other HPV-associated malignancies [9]. They reported higher methylation in HGAIN and anal cancer for two genes compared to normal mucosa or low-grade lesions suggesting a role for DNA methylation in anal carcinogenesis. Additional epigenetic targets unique to anal cancer may be uncovered by a more comprehensive high-throughput methylation array approach. However, due in part to the limited quantity and quality of anal cancer tissue specimens, broad scale genomic techniques have not been widely applied to this disease site. The successfully met objectives of this study were first, to demonstrate the feasibility of investigating DNA methylation in anal cancer utilizing methylation array technology and second, to identify CpG loci that were differentially methylated in invasive SCC compared to pre-invasive and/or normal mucosa.

## Materials and Methods

### Ethics Statement

Our study was approved by the Institutional Review Board at the University of South Florida as exempt and not requiring informed consent from study subjects. Data were collected and appropriately de-identified prior to analysis.

### Case Identification and Tissue Collection

The records of all patients treated at the H. Lee Moffitt Cancer Center and Research Institute from 2000–2008 with the diagnosis of anal SCC or SCC *in situ* (SCC-IS) were reviewed. Patients with a pathological diagnosis of SCC or SCC-IS of the anus and sufficient formalin-fixed paraffin-embedded (FFPE) tissue for analysis were identified. Pertinent clinical data were collected retrospectively utilizing our institutional electronic medical record system. We identified 24 patients treated at the Moffitt Cancer Center that met our inclusion criteria. Median age for the 24 patients (13 females and 11 males) in the study population was 44 years (range 26–81). Five patients were immunocompromised secondary to HIV (n = 3) or immunosuppressive medications given for organ transplantation (n = 2).

To ensure the accuracy of diagnosis, tissue samples were re-reviewed and regions of histologically normal mucosa, SCC-IS, and SCC were marked by a dedicated gastrointestinal pathologist (DC). FFPE tissues were subsequently cut (15 µm thick) and meticulously macrodissected to reduce cross contamination. Prior to the macrodissection of each case, gloves and instruments were changed and the workspace was disinfected. Of note, for the purposes of this study, SCC-IS is considered equivalent to AIN III and HGAIN.

### HPV Genotyping

DNA was extracted from FFPE tissues using QIAamp DNA FFPE Tissue Kit (Qiagen Inc, Valencia, CA). HPV genotyping was performed using the INNO-LiPA HPV Genotyping *Extra kit* (Innogenetics, Belgium). In brief, 100 ng of DNA was utilized for PCR amplification of a short fragment (65-bp) of the HPV L1 region with biotinylated primers (SPF_10_) using the MJ PTC-200 DNA engine thermocycler. PCR products were hybridized to the AutoBlot 3000H 20 Strip, a probe-specific nitrocellulose test strip, placed on an adhesive LiPA-Scan Reading template and analyzed using the LiRAS for LiPA HPVE v2.01 software (Innogenetics, Belgium). All assays included the amplification of a 270 bp fragment of HLA-DPB1 as a positive control for human DNA. All PCR runs met quality control standards, with all samples positive for internal positive controls and negative controls negative for each run. This system detects 28 HPV types (HPV 6, 11, 16, 18, 26, 31, 33, 35, 39, 40, 43, 44, 45, 51, 52, 53, 54, 56, 58, 59, 66, 68, 70, 73, and 82). High-risk HPV types were defined as the 12 high-risk types classified as group 1 carcinogens (16, 18, 31, 33, 35, 39, 45, 51, 52, 56, 58, and 59) [17].

### Methylation Array

Genomic DNA (500 ng) was sodium bisulfite-modified using the EZ DNA Methylation kit (Zymo Research, Orange, CA) following the manufacturer’s instructions. DNA methylation was measured using the Illumina GoldenGate methylation assay with the Cancer Panel 1 probes (Illumina, San Diego, CA) following standard protocols [18]. This bead array platform interrogated 1505 CpG loci that represent 807 cancer-related genes. In brief, following methylation-specific hybridization, allele specific oligonucleotides were extended and ligated to a locus-specific oligonucleotide (LSO), which served as the template for fluorescently labeled universal primers that amplify either unmethylated (U) or methylated (M) templates. Labeled DNA contained a unique IllumiCode address for hybridization to its complement bead type (∼30 replicates per CpG site) on a Sentrix Array Matrix (SAM) plate. At each CpG site, U and M fluorescent intensities were measured, averaged across replicate beads, and compared to a panel of negative controls using the Illumina’s BeadStudio Methylation Module v3.2. Probes that were significantly different from negative controls were included in the analysis.

### Bisulfite Sequencing

#### Primer design and amplification of target CpG site

Primer sets targeting *HOXA5, TGFβ3*, and *KRT1* genes were designed with MethPrimer software [19] or derived from the published literature (**Table 1**). The primers specifically amplified the CpG sites measured in the Illumina GoldenGate assay. Using 2 µl of bisulfite-converted DNA as PCR template, a 50 µl reaction containing 0.02 µM of each primer, 0.2 mM dNTP, 1 unit of HotStarTaq Plus polymerase and varying amounts of magnesium was performed. Initial denaturation was at 95°C for 15 min, followed by 40 cycles of 94°C for 1 min, 48.2°C–56.5°C annealing for 1 min and 72°C for 1 min; and a final extension cycle of 72°C for 10 min. PCR products were viewed with a 1.5% agarose gel stained with ethidium bromide. Target amplicons were gel-extracted and purified using the QIAquick Gel Extraction Kit (Qiagen Inc., Valencia, CA) according to the manufacturer’s instructions.

**Table 1:** Primers for Bisulfite Sequencing,

Gene	Forward Primer	Reverse Primer	Annealing Temp	MgCl2	Amplicon size (bp)
*HOXA5*	TTA TTA GGA TGT ATT AAT TGT TAG GT	CAA AAT TCA AAA CTA CTA ACA AAA C	48.2	2.5	192
*TGFB3*	GAT TGA GGT TTG GTA AGA AGG TGT A	ACT AAA AAT CAA AAC CCA ACA AAA C	56.5	2.5	167
*KRT1*	TAG AGT AGG AGA TAG ATA TTA G	TCC AAT ATA AAA CTT AAA TCA CC	48.2	2.75	176

#### Cloning, transformation, and sequencing

Gel-purified PCR products were ligated into a TA cloning vector, pCR® 4 -TOPO®, using Invitrogen’s TOPO cloning kit (Life Technologies, Carlsbad, CA), transformed into *Escherichia coli* competent cells, and plated on 100 mg/ml LB-ampicillin agar plates. Ligation and transformation was confirmed by PCR (12.50 µl of Platinum Supermix, 0.5 µl each of 10 µM gene-specific forward primer, M13 Universal Reverse primer and colony template) using the following thermocycler conditions: 1 cycle at 95°C for 6 min; 40 cycles of 94°C for 30 secs, appropriate annealing temperature (**Table 1**) for 30 secs and 72°C for 1 min, and 1 cycle at 72°C for 10 min. PCR confirmed positive colonies were inoculated into LB-Amp broth and incubated overnight at 37°C. Plasmid DNA was extracted using the QIAGEN Miniprep Kit (Qiagen Inc., Valencia, CA) according to the manufacturer’s instructions. Plasmid DNA was sequenced using M13 universal primers on the ABI 3600 sequencer (Applied Biosystems, Foster City, CA).

### Methylation Array Bioinformatics and Statistical Analyses

**Figure 1:**
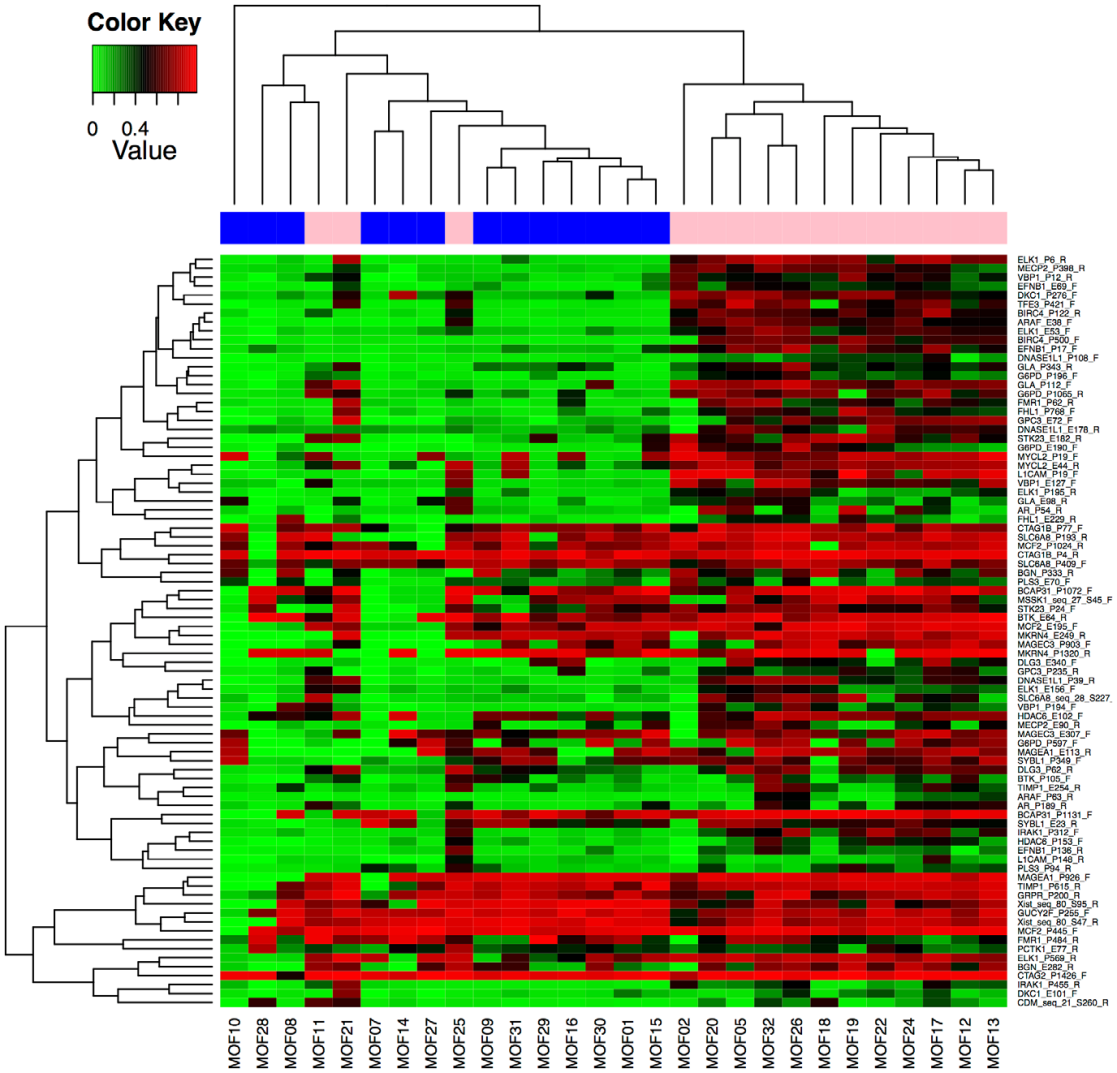
Gender Heatmap of X-linked CpG Loci. DNA methylation is involved in the transcriptional activation of genes on one of the two X chromosomes in female somatic cells. In general, male and female cases clustered together with methylation levels of the majority of X-linked genes correlating well with the gender of the tissue source (i.e. little-to-no methylation in male samples and hemi-methylation in female samples). Sample gender is represented above the heatmap (pink = female, blue = male). Methylation is represented by the beta value, or percent of total signal with green representing low methylation and red representing hemi-methylation. 51 of 84 loci within X-linked genes were differentially methylated (p<0.05) between males and females.

Methylation data were pre-processed by setting non-detected probes as N/A (Not Applicable) for samples in which the detection p value for the probe was >0.05. Chip-wide controls and Multi-Dimensional Scaling plots were used to visualize data quality. Methylation data were analyzed using the R statistical software package and Bioconductor packages. Internal functional validation of the assay was performed by unsupervised clustering to confirm separation by gender due to the presence of X-linked CpG sites on the array **(** **Figure 1** **)** [18]. Analysis of X-linked genes with respect to gender was performed using the Mann-Whitney non-parametric test with p<0.05 as the threshold for significance. Clustering was performed using non-centered correlation as the similarity metric within R. X-linked methylation probes were then discarded prior to further analysis.

The number of paired, matched tissues was small; therefore, we examined the three groups of tissues in a pooled fashion to identify targets with differential methylation in pre-invasive (normal and SCC-IS) vs. invasive cancer samples. Due to non-normal distribution of beta ratios, we applied a Mann-Whitney test for differences in groups to a total of 1,421 CpG loci (1,505–84 X-linked loci) and selected significant loci using a conservative threshold to partially correct for multiple testing problems (p<0.01). Boxplots were used to visualize aggregate methylation data, representing the median methylation of each group. Additional analysis was performed using a Kruskal-Wallis non-parametric test to accommodate variables with three levels (Normal, SCC-IS and Invasive).

## Results

### HPV Genotyping

HPV genotyping was performed on adjacent histologically normal anal mucosa (n = 3), SCC-IS (n = 11), and SCC (n = 15) tissues. HPV viral DNA was detected in all tissues, with 38 total infections detected (33 high-risk and 5 low-risk types, **Table 2** **)**. High-risk HPV types were detected in 93% (27/29) of tissues; 3 patients with SCC-IS were positive for low-risk HPV 6. HPV 16 was the most common infection, being detected in 76% (22/29) tissue samples overall and in 87% of SCCs. It was most often detected as a single infection (59%, 17/29 tissue sections). Two SCCs were positive for HPV 52; whereas HPV 18 was only detected in one SCC-IS. Infection with multiple HPV types was detected in 7 (29%) of 24 patients, of which two were HIV positive. The detection of low-risk and high-risk types occurred concurrently in 3 out of the 5 low-risk infections detected; specifically, two SCC tissues had co-infection with HPV 11 and 16. Of the SCC-IS tissues where HPV 6 was detected, only one had a concurrent high-risk type detected (HPV 52). No correlation between multiple infections and histological diagnosis (normal mucosa, SCC-IS, SCC) or immunocompetency was identified.

**Table 2:** HPV Genotyping of Anal Tissues: Overall Prevalence and Across the Spectrum of Anal Neoplasia.

HPV Type	Overall HPV Prevalence* (n = 24 Patients)	Normal Mucosa (n = 3 Tissues)	SCC-in Situ (n = 11 Tissues)	SCC (n = 15 Tissues)
**High Risk**				
** HPV-16**	22 (92%)	3 (100%)	6 (54%)	13 (87%)
** HPV-18**	1 (4%)	–	1 (9%)	–
** HPV-31**	1 (4%)	–	1 (9%)	–
** HPV-39**	2 (8%)	–	1 (9%)	1 (7%)
** HPV-52**	6 (25%)	–	4 (36%)	2 (13%)
** HPV-54**	1 (4%)	–	1 (9%)	–
**Low Risk**				
** HPV-6**	3 (13%)	–	3 (27%)	–
** HPV-11**	2 (8%)	–	–	2 (13%)

* A total of 38 HPV infections were detected in 29 tissues, due to multiple infections**.**

### Quality Control Analysis of GoldenGate DNA Methylation Data

The GoldenGate array contains standard control probes that assess several parameters including 1) Allele specific extension, 2) Bisulfite Conversion, 3) Extension Gap, 4) First hybridization, 5) Gender, 6) Negative Control, 7) PCR Contamination, and 8) Second Hybridization [18]. All cases used for this study were reviewed and passed these quality control measures. **Figure 1** presents the quality control analysis of the methylation probes by gender to confirm separation due to the presence of X-linked CpG sites on the array [18]. In an unsupervised cluster analysis of X-linked CpG sites, a distinct separation of patients by gender was observed with 51 of 84 (61%) X-linked genes found to be differentially methylated (p<0.05) between specimens derived from male and female patients. These analyses support the internal validity of our methylation data.

### Differential Methylation in Invasive SCC vs. Pre-invasive Anal Tissue

**Figure 2:**
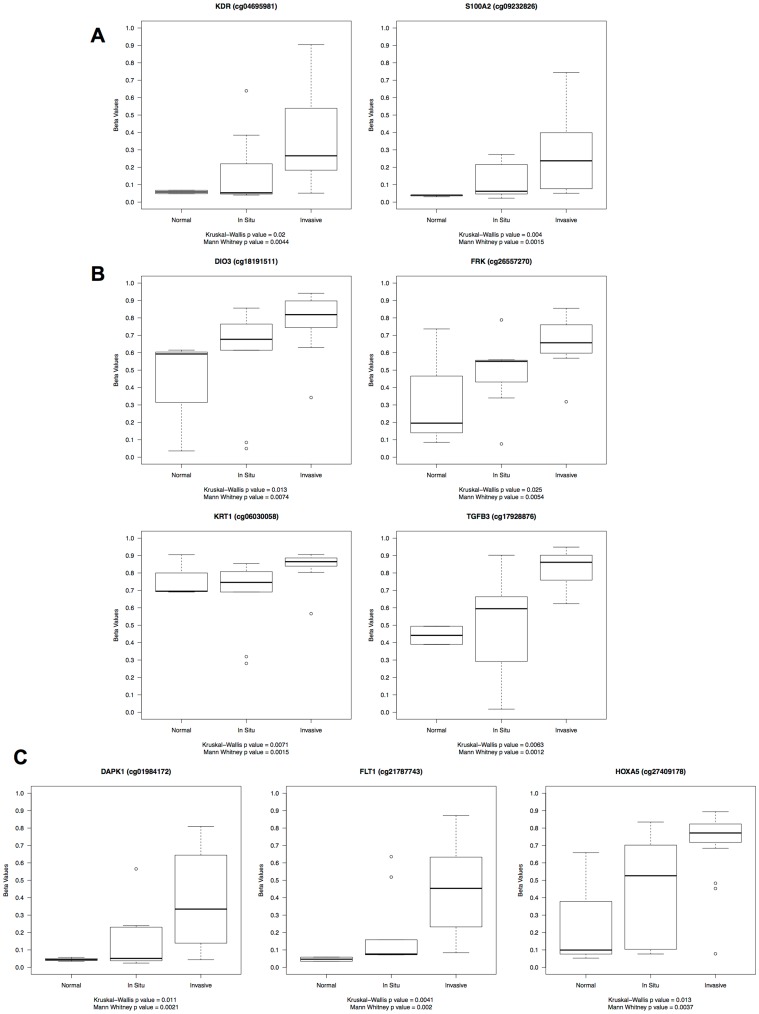
Boxplot Representations of Selected Differentially Methylated CpG Loci. Boxplots illustrating methylation levels of selected significant differentially methylated CpG sites (Mann-Whitney p<0.01) across histologic subtypes (normal and SCC-IS vs. invasive CC). Three patterns are identified: A: Loci with low methylation levels (median beta value <0.3) across all tissues. B: Loci with high methylation levels (median beta value ≥0.3) across all tissues. C: Loci demonstrating little to no methylation in non-invasive tissues with significant levels of methylation in invasive SCC.

To optimize the numbers of cases per comparison group, methylation profiles were compared between pre-invasive anal tissue (normal and SCC-IS) and invasive SCC specimens using the Mann-Whitney test. A total of 22 CpG loci corresponding to 20 genes was noted to have significant (p<0.01) differential methylation between the two groups (**Table 3**). Of these, all but 2 CpG loci demonstrated increased methylation in invasive SCC compared to pre-invasive tissues (**Figure 2** and **Figure S1**)**.** Methylation levels of these 2 CpG sites both within the *GABRA5* gene were significantly lower in invasive SCC compared to pre-invasive tissues. (**Table S1** provides detailed annotation for genes containing differentially methylated CpG loci).

**Table 3:** Genes with Differentially Methylated CpG Loci in the Progression of Anal Neoplasia.

Symbol	Product	Annotation	CpG Number*	P-value	
				MW†	KW‡
**Growth Regulation and Cell Cycle Control**					
*TGFβ3*	Transforming growth factor,beta 3	Controls proliferation and differentiation	cg17928876	0.0012	0.0063
*FRK*	Fyn-related kinase	SRC kinase family with epithelial tissue-specificexpression	cg26557270	0.0054	0.0247
*PADI4*	Peptidyl arginine deiminase,type IV	Post-translational modification (arginine methylationand citrull-ination) of histones	cg19159961	0.0030	0.0155
*ID1*	Inhibitor of DNA binding 1 isoform a	Helix-loop-helix protein	cg09569033	0.0071	0.0228
**Differentiation**					
*S100A2*	S100 calcium binding proteinA2	Small, acidic Ca(2+)-binding proteins in nucleus	cg09232826; cg21074565	0.0015;0.0083	0.0040; 0.0325
*KRT1*	Keratin 1	Differentiation-dependent keratin	cg06030058	0.0015	0.0071
*KRT5*	Keratin 5	Primary keratin	cg04254916	0.0033	0.0154
*PRSS8*	Prostasin pre-protein	Trypsinogen; serine proteases	cg27436259	0.0022	0.0116
**Angiogenesis**					
*FLT1*	Fms-related tyrosine kinase 1	VEGF receptor tyrosine kinase (also VEGFR1)	cg21787743	0.0020	0.0041
*KDR*	Kinase insert domain receptor	VEGF Type III receptor tyrosine kinase (also VEGFR2)	cg04695981	0.0044	0.0197
**Apoptosis**					
*DAPK1*	Death-associated protein kinase 1	Calmodulin-dependent serine-threonine kinase	cg01984172	0.0021	0.0106
*HOXA5*	Homeobox A5	DNA-binding transcription factor	cg27409178	0.0037	0.0133
*TNFRSF10B*	Tumor necrosis factor receptor superfamily, 10b	Death domain associated receptor	cg07508317	0.0026	0.0112
*BCL2A1*	BCL2-related protein A1	Reduces pro-apoptotic cytochrome C release; blocks caspase activation.	cg27177709	0.0061	0.0262
*SEMA3B*	Semaphorin 3B isoform 1	Extracellular secreted protein important in axonal guidance; induces apoptosis	cg12999941	0.0083	0.0283
**Other Processes**					
*CCL3*	Chemokine (C-C motif) ligand 3	Macrophage inflammatory protein-1	cg05481196	0.0096	0.0374
*P2RX7*	Purinergic receptor P2X7isoform b	Cell surface ATP receptor; ligand-gated ion channel	cg08688169	0.0096	0.0156
*CD9*	CD9 antigen	Cell surface tetraspanin (TM4SF) glycoprotein	cg19415774	0.0071	0.0228
*DIO3*	Deiodinase, iodothyronine, III	Selenoenzyme	cg18191511	0.0074	0.0133
*GABRA5*	Gamma-aminobutyric acid A receptor, alpha 5	heteromeric pentameric ligand-gated ion channels	cg02225257; cg20051555	0.0030; 0.0044	0.0112; 0.0044

* CpG locus label within the GoldenGate methylation array.

† Mann-Whitney test for methylation differences between two groups (pre-invasive vs. invasive SCC) using a conservative threshold to partially correct for multiple testing problems (p<0.01).

‡ Kruskal-Wallis non-parametric test performed to test three levels (Normal, *SCC-IS* and Invasive).

There were three distinct patterns of methylation observed in the differentially methylated CpG panel (**Figure 2**): 1) Loci with low methylation levels (median beta value <0.3) in all tissue types (e.g. *ID1, KDR, TNFRSF10B, and SEMA3B*) but with a statistically significant difference between non-invasive and invasive tissues; 2) loci with high methylation levels (median beta ≥0.3) in all tissue types (e.g. *GABRA5*, *BCL2A1, CCL3, DI03, FRK, KRT1, KRT5, P2RX7, PRSS8,* and *TGFB3*) but with a statistically significant difference between non-invasive and invasive tissues; and 3) loci with little to no methylation in non-invasive tissues (beta values<0.3) with a significant increase above the threshold into high methylation levels (beta ≥0.3) in invasive SCC (e.g. *CD9, DAPK1, FLT1, HOXA5,* and *PADI4*). For S100A2, one CpG site demonstrated low methylation levels in all tissues (median beta value<0.3) while the other was consistent with pattern 3; however, the general trend was suggestive of a low to high methylation pattern across both sites within this gene.

Although the number of samples in each group was relatively small, and particularly, in the normal tissue group, we performed an exploratory analysis to identify targets with differential methylation across all three groups (normal, SCC-IS and SCC). Using a Kruskal-Wallis non-parametric test and p-value <0.01, 7 probes had differential methylation across the 3 groups, four of which (*S100A2, FLT1, TGFB3* and *KRT1*) were identified in our binary analysis (**Table 3** and **Figure 2**) and three were not (*BMP4, EV12A, and IL1RN*). In fact, the top 20 significant and near significant (p<0.02) differentially methylated loci from the Kruskal-Wallis analysis and the top 20 significant (p<0.01) loci from the Mann-Whitney analysis were highly similar with 13 CpG sites in common.

### Measurement of Methylated Targets by Bisulfite Sequencing

Given the scarcity of these specimens, a confirmatory measurement of methylation using conventional bisulfite sequencing was performed in a total of 9 anal tissues (8 SCC and 1 SCC-IS) for which there was sufficient remaining tissue and/or genomic DNA. We selected 3 genes for the determination of methylation status by bisulfite sequencing: *TGFβ3* and *KRT1* had high methylation in all tissue types while *HOXA5* demonstrated a progression of methylation from low to high levels across tissues. Within this study, the median beta values for CpG sites within *TGFβ3, KRT1* and *HOXA5* genes were 0.86, 0.86 and 0.77, respectively. Within the set of 9 patients, the average methylation levels at the CpG site of interest, as determined by bisulfite sequencing, for *TGFβ3, KRT1* and *HOXA5* were very similar to the corresponding average array beta values (63% vs. 69%; 68% vs. 76% and 63% vs. 63%, respectively). On an individual tissue specimen level, we demonstrated evidence of methylation (median beta value of ≥0.3) at the CpG site of interest in ≥30% of corresponding clones by bisulfite sequencing at concordance rates of 88% (8 of 9 clones) for *TGFβ3*, 75% (6/8) for *HOXA5* and 100% (9/9) for *KRT1*. In all cases of non-concordance, a low array beta value was associated with a higher percentage of methylated clones by sequencing. Our findings confirm previous reports of high concordance between GoldenGate methylation array technology and conventional methylation assays [20], [21].

## Discussion

The molecular events involved in HPV-associated anal carcinogenesis, including alterations in host genome methylation, remain poorly studied. With respect to HPV characterization, all patients in our study were found to be infected with one or more HPV genotypes. HPV 16 was the predominant oncogenic subtype identified; however, HPV 18, 32, 39 and 52 were also observed. The HPV prevalence and type distribution are similar to previous reports in anal cancer [22]–[26]. In the current investigation, we utilized an FFPE-tissue compatible methylation array to evaluate the methylation status of 1,505 CpG loci representing 807 genes. We identified a panel of 20 CpG loci representing 19 genes with increased methylation in the progression from pre-invasive normal and SCC-IS tissues to invasive anal SCC. Of these CpG loci, 6 had low methylation levels in normal and pre-invasive tissues but were hypermethylated in invasive SCC. From a biological standpoint, this panel of CpG sites may represent biomarkers of anal neoplastic progression from non-invasive to invasive anal neoplasia and may reflect carcinogenesis-related epigenetic alterations with a transition from an unmethylated to methylated state.

We identified a panel of CpG loci that were differentially methylated in HPV-associated anal SCC (**Table 3** and **Table S1**). These epigenetic events occurred in genes encoding proteins that play a role in growth regulation/cell cycle control (e.g. TGF-B3[27]–[29], FRK[30]–[32], PADI4[33]–[36], and ID[37]–[41]) and critical regulation of apoptosis (e.g. TNFRSF10B[42]–[47], DAPK1[9], [48]–[50], HOXA5 [51]–[54], BCL2A1[55]–[59], and SEMA3B[60]–[62]). CpG loci that were shown to have high methylation levels across the spectrum of anal tissues but with a significant difference between non-invasive tissues and invasive SCC may be markers of early epigenetic alterations in HPV-induced carcinogenesis. For example, methylation of CpG loci in two keratin genes, *KRT1* and *KRT5*, the products of which contribute to the differentiation of squamous epithelium, may be early biomarkers of HPV-associated carcinogenesis[63]–[65]. The HPV oncogenes E6 and E7 primarily target p53 and Rb, respectively, for degradation [66]. Interestingly, methylation levels of several CpG loci occurred within genes that either interact with or are part of the p53 or Rb pathways, including *PAD14* [33], [35], *HOXA5* [67], *FRK* [32], and *DAPK1* [50] (**Table S1**). Several differentially methylated loci identified in this study appear within genes that may be potential novel targets in the setting of cancer, such as *FRK, BCL2A1, GABRA5, DIO3, P2RX7, CCL3* and *ID*. Overall, there appears to be a clustering of methylation in CpG sites across genes with similar functions within anal SCC and these epigenetic differences may have potential utility as biomarkers of HPV-associated carcinogenesis (**Table S1**) [68]–[93].

Zhang et al. evaluated the methylation status of 11 candidate genes (*DAPK1, IGSF4, MLH1, HIC1, RARB, p14, TP73, MGMT, RASSF1, APC,* and *CDKN2A*) in a set of 172 anal biopsies, which is the only published analysis of DNA methylation in anal cancer [9]. They reported an increased frequency of *DAPK1* and *IGSF4* methylation during the progression from normal mucosa to SCC-IS to invasive SCC. In our study, we have indeed confirmed that CpG loci within *DAPK1* were among the most significantly methylated sites with increasing methylation across the spectrum of anal carcinogenesis. However, neither of the *IGSF4* probes demonstrated significant differential methylation in our study (p = 0.029 and 0.052 respectively by Mann-Whitney, data not shown). While data are limited in anal cancer, there is evidence for aberrant DNA methylation in HPV-associated cervical cancer [5], [9], [94]. Of the significant differentially methylated sites identified in our anal cancer study, *DAPK1* is the only methylation target that has been similarly shown to be methylated in invasive cervical SCC (55%) and high-grade pre-invasive lesions (52%), with reduced methylation in normal and low-grade lesions [48], [49], [95]–[98]. These data support a possible role for the epigenetic alteration of *DAPK1* in HPV-associated carcinogenesis.

Until recently, the mechanisms responsible for the association between HPV infection and epigenetic alterations have been somewhat speculative with limited *in vitro* data. HPV has been shown to upregulate and augment DNMT [14], [15], [99], [100] and HDAC activity [14], [16]; thus it is biologically plausible that epigenetic alterations may play a role in HPV-induced carcinogenesis. Leonard et al. [15] have demonstrated that the transfection of the episomal forms of both HPV16 and 18 result in the induction of DNMT1 and DNMT3B expression and subsequent alterations in methylation status of numerous host genes across the genome. Of interest, they observed that the majority of HPV-induced methylation targets appeared to be non-random and were associated with cis-acting events (e.g. increased CpG dinucleotide density, CpG sites near telomeres and known HPV integration sites) and clustered across genes within specific chromosomal locations (e.g. HPV methylation hotspots) [15]. We determined that 19 of the 22 differentially methylated CpG loci identified in our study occurred in genes that fit the criteria for these “methylation-prone” areas (**Table 4**). *TGFB3, GABRA5, KDR, FRK* and *CCL3* all localize directly to known HPV integration sites while *BCL2A1* is immediately adjacent to one such site [15], [101]–[105]. *HOXA5* is located at an exact HPV 16 and 18 hypermethylation hotspot while *DAPK1, DIO3,* and *P23X7* are all immediately adjacent to a described hotspot [15]. *FLT1, PRSS8* and *ID1* are located in the telomeric regions of their respective chromosomes. Finally, we have noted what appears to be a novel hotspot at 12q13.3 with *KRT1, KRT5* and *CD9* all mapping directly to this locus. Our findings lend further support to the notion that HPV-associated methylation events appear to occur in a non-random fashion and suggest that there may be similarities in epigenetic alterations across HPV-associated cancers. A critical area for future research will be to distinguish between epigenetic alterations that are oncogenic drivers versus those that may simply be bystander effects of HPV infection.

**Table 4:** Chromosomal Mapping of Genes with Differentially Methylated CpG Loci.

Gene Symbol	Chromosomal Location	Relation to HPV or hotspots
*TGFB3*	14q24.3	HPV integration site
*GABRA5*	15q12	HPV integration site
*FRK*	6q22.1	HPV integration site
*CCL3*	17q12	HPV integration site
*KDR*	4q12	HPV integration site
*BCL2A1*	15q25.1	Close to 15q25.3 HPV integration site
*HOXA5*	7p15.2	HPV16 and 18 hypermethylation hotspot
*DAPK1*	9q21.33	Close to 9q21.31 HPV18 hypermethylation hotspot
*DIO3*	14q32.31	Close to 14q32.33 HPV 16 and 18 hypermethylation hotspot
*P2RX7*	12q24.31	Close to 12q24.33 HPV 16 and 18 hypermethylation hotspot
*S100A2*	1q21.3	Close to 1q21.1 HPV16 and 18 hypomethylation hotspot
*KRT1*	12q13.3	Potential novel hypermethylation hotspot with KRT5 and CD9
*KRT5*	12q13.3	Potential novel hypermethylation hotspot with KRT1 and CD9
*CD9*	12q13.3	Potential novel hypermethylation hotspot with KRT1 and KRT5
*PRSS8*	16p11.2	Telomeric region
*ID1*	20q11.2	Telomeric region
*FLT1*	13q12.2–12.3	Telomeric region
*TNFRSF10B*	8p21.3	No associations
*SEMA3B*	3p21.31	No associations
*PADI4*	1p36.13	No associations

To our knowledge, this study is the first to demonstrate the feasibility of a large-scale evaluation of DNA methylation in FFPE tissues from anal SCC, which in addition includes corresponding HPV genotyping. We acknowledge the fact that the GoldenGate array interrogates a number of selected known methylation targets and as such, does not represent a full evaluation of genome-wide events. In addition, with this array, we were unable to determine whether differences in methylation at specific CpG loci had biological consequences, such as gene silencing. However, differential methylation events at specific loci may still represent potential biomarkers of HPV-associated carcinogenesis. This study is certainly limited by its small sample size, which unfortunately is inherent with the relative paucity of anal cancer tissues treated at one center. Normal anal mucosa was identified by histologic review; however, these tissues were HPV-positive and adjacent to SCC-IS or SCC, thus, do not represent purely unaffected anal mucosa. Non-neoplastic anal tissues infected by HPV may have altered epigenetic profiles but nonetheless are relevant as the earliest end of the spectrum of HPV-associated anal carcinogenesis. In this study, due to a small sample size, we were unable to fully analyze differences in methylation by HPV genotype. However, in an exploratory analysis, we identified a panel of 27 CpG loci that were differentially methylated (p<0.05 by Mann Whitney) between the HPV16+ and HPV16- tissues (data not shown). Larger studies are needed to fully evaluate the impact of HPV-related factors, such as genotype or number of infections, on epigenetic profiles and associated biologic pathways.

Our data provide preliminary evidence that the progression from normal anal mucosa and anal SCC-IS to invasive SCC is associated with distinct alterations in host genome methylation which in turn may be a consequence of high risk HPV infection. With the application of broad high-throughput methylation profiling, we have generated a candidate list of progressively methylated CpG loci across the spectrum of anal squamous neoplasia, including sites within genes previously unassociated with anal SCC. Despite the small numbers of patients evaluated in this study, our findings are in agreement with what is known about the impact of HPV on epigenetic profiles including increased methylation at or close to specific chromosomal locations including previously described HPV methylation “hotspots” and viral integration sites. Our work represents an important initial step in understanding the epigenetic events associated with anal carcinogenesis. A critical translational step would be to apply broad methylation assays to identify a relevant panel of methylation biomarkers that could not only refine the diagnosis of HGAIN or SCC-IS but also serve as an indicator for increased risk of subsequent malignant transformation to invasive SCC. Molecular guidance ultimately may reduce the burden of potentially unnecessary screening, biopsy and treatment procedures. Further evaluation of methylation events in anal squamous neoplasia is clearly warranted in larger patient populations and may contribute not only to a better understanding of anal carcinogenesis, but also to the development of novel biomarkers that may ultimately impact on enhanced diagnosis, screening and prevention.

## Supporting Information

Figure S1 **Boxplot Representations of All Differentially Methylated Loci.** Boxplots illustrating methylation levels of 22 significant differentially methylated CpG sites representing 20 genes (Mann-Whitney p<0.01) across histologic subtypes (normal and SCC-IS vs. invasive CC). Three patterns are identified: A: Loci with low methylation levels (median beta value <0.3) across all tissues. B: Loci with high methylation levels (median beta value ≥0.3) across all tissues. The two CpG sites within the *GABRA5* gene demonstrated reduced methylation with neoplastic progression C: Loci demonstrating little to no methylation in non-invasive tissues with significant levels of methylation in invasive SCC.
(TIF)

Table S1 **Genes with Differentially Methylated CpG Loci in the Progression of Anal Neoplasia with Full Annotation.**
(DOC)

## References

[1] American Cancer Society (2012) Cancer Facts & Figures 2012. Atlanta, Ga.

[2] Howlader N, Noone AM, Krapcho M, Neyman N, Aminou R, et al.. (2012) SEER Cancer Statistics Review, 1975–2009 (Vintage 2009 Populations). National Cancer Institute. Bethesda, MD.

[3] Eng C (2006) Anal cancer: current and future methodology. Cancer Invest 24: 535–544. 16939964 10.1080/07357900600815208

[4] International Agency for Research on Cancer (2007) IARC Monographs on the Evaluation of Carcinogenic Risks to Humans, Volume 90 Human papillomaviruses; WHO and IARC, editors. Lyon, France. 1–636 p. PMC4781057 18354839

[5] Whiteside MA, Siegel EM, Unger ER (2008) Human papillomavirus and molecular considerations for cancer risk. Cancer 113: 2981–2994. 18980282 10.1002/cncr.23750

[6] Scholefield JH, Harris D, Radcliffe A (2011) Guidelines for management of anal intraepithelial neoplasia. Colorectal Dis 13 Suppl 1 3–10. 10.1111/j.1463-1318.2010.02494.x 21251167

[7] Darragh TM, Winkler B (2011) Anal cancer and cervical cancer screening: key differences. Cancer Cytopathol 119: 5–19. 21319310 10.1002/cncy.20126

[8] Grulich AE, Jin F, Conway EL, Stein AN, Hocking J (2010) Cancers attributable to human papillomavirus infection. Sex Health 7: 244–252. 20719211 10.1071/SH10020

[9] Zhang J, Martins CR, Fansler ZB, Roemer KL, Kincaid EA, et al (2005) DNA methylation in anal intraepithelial lesions and anal squamous cell carcinoma. Clin Cancer Res 11: 6544–6549. 16166431 10.1158/1078-0432.CCR-05-0374

[10] Gervaz P, Hahnloser D, Wolff BG, Anderson SA, Cunningham J, et al.. (2004) Molecular biology of squamous cell carcinoma of the anus: a comparison of HIV-positive and HIV-negative patients. J Gastrointest Surg 8: 1024–1030; discussion 1031. 10.1016/j.gassur.2004.08.013 15585390

[11] Zucchini C, Concu M, Martini F, Morelli C, Salfi N, et al (2007) FHIT oncosuppressor gene expression profile in human anal cancers. Int J Biol Markers 22: 39–42. 17393360 10.1177/172460080702200106

[12] Jones PA, Baylin SB (2002) The fundamental role of epigenetic events in cancer. Nat Rev Genet 3: 415–428. 12042769 10.1038/nrg816

[13] Turan T, Kalantari M, Calleja-Macias IE, Cubie HA, Cuschieri K, et al (2006) Methylation of the human papillomavirus-18 L1 gene: a biomarker of neoplastic progression? Virology 349: 175–183. 16472835 10.1016/j.virol.2005.12.033

[14] Burgers WA, Blanchon L, Pradhan S, de Launoit Y, Kouzarides T, et al (2007) Viral oncoproteins target the DNA methyltransferases. Oncogene 26: 1650–1655. 16983344 10.1038/sj.onc.1209950 PMC3350866

[15] Leonard S, Wei W, Collins S, Pereira M, Diyaf A, et al (2012) Oncogenic Human Papillomavirus imposes an instructive pattern of DNA methylation changes which parallel the natural history of cervical HPV infection in young women. Carcinogenesis 33: 1286–1293. 22552403 10.1093/carcin/bgs157

[16] Avvakumov N, Torchia J, Mymryk JS (2003) Interaction of the HPV E7 proteins with the pCAF acetyltransferase. Oncogene 22: 3833–3841. 12813456 10.1038/sj.onc.1206562

[17] Schiffman M, Clifford G, Buonaguro FM (2009) Classification of weakly carcinogenic human papillomavirus types: addressing the limits of epidemiology at the borderline. Infect Agent Cancer 4: 8. 19486508 10.1186/1750-9378-4-8 PMC2694995

[18] Bibikova M, Lin Z, Zhou L, Chudin E, Garcia EW, et al (2006) High-throughput DNA methylation profiling using universal bead arrays. Genome Res 16: 383–393. 16449502 10.1101/gr.4410706 PMC1415217

[19] Li LC, Dahiya R (2002) MethPrimer: designing primers for methylation PCRs. Bioinformatics 18: 1427–1431. 12424112 10.1093/bioinformatics/18.11.1427

[20] McRonald FE, Morris MR, Gentle D, Winchester L, Baban D, et al (2009) CpG methylation profiling in VHL related and VHL unrelated renal cell carcinoma. Mol Cancer 8: 31. 19493342 10.1186/1476-4598-8-31 PMC2698845

[21] Milani L, Lundmark A, Kiialainen A, Nordlund J, Flaegstad T, et al (2010) DNA methylation for subtype classification and prediction of treatment outcome in patients with childhood acute lymphoblastic leukemia. Blood 115: 1214–1225. 19965625 10.1182/blood-2009-04-214668

[22] Sobhani I, Walker F, Roudot-Thoraval F, Abramowitz L, Johanet H, et al (2004) Anal carcinoma: incidence and effect of cumulative infections. Aids 18: 1561–1569. 15238774 10.1097/01.aids.0000131335.15301.dd

[23] Grulich AE, Jin F, Conway EL, Stein AN, Hocking J (2010) Cancers attributable to human papillomavirus infection. Sex Health 7: 244–252. 20719211 10.1071/SH10020

[24] Wong AK, Chan RC, Aggarwal N, Singh MK, Nichols WS, et al (2010) Human papillomavirus genotypes in anal intraepithelial neoplasia and anal carcinoma as detected in tissue biopsies. Mod Pathol 23: 144–150. 19838162 10.1038/modpathol.2009.143

[25] Noffsinger A, Witte D, Fenoglio-Preiser CM (1992) The relationship of human papillomaviruses to anorectal neoplasia. Cancer 70: 1276–1287. 1324782 10.1002/1097-0142(19920901)70:3+<1276::aid-cncr2820701514>3.0.co;2-b

[26] Fenger C, Frisch M, Jass JJ, Williams GT, Hilden J (2000) Anal cancer subtype reproducibility study. Virchows Arch 436: 229–233. 10782881 10.1007/s004280050035

[27] Archey WB, Sweet MP, Alig GC, Arrick BA (1999) Methylation of CpGs as a determinant of transcriptional activation at alternative promoters for transforming growth factor-beta3. Cancer Res 59: 2292–2296. 10344731

[28] Nees M, Geoghegan JM, Munson P, Prabhu V, Liu Y, et al (2000) Human papillomavirus type 16 E6 and E7 proteins inhibit differentiation-dependent expression of transforming growth factor-beta2 in cervical keratinocytes. Cancer Res 60: 4289–4298. 10945644

[29] Murvai M, Borbely AA, Konya J, Gergely L, Veress G (2004) Effect of human papillomavirus type 16 E6 and E7 oncogenes on the activity of the transforming growth factor-beta2 (TGF-beta2) promoter. Archives of virology 149: 2379–2392. 15290353 10.1007/s00705-004-0376-x

[30] Cance WG, Craven RJ, Bergman M, Xu L, Alitalo K, et al (1994) Rak, a novel nuclear tyrosine kinase expressed in epithelial cells. Cell Growth Differ 5: 1347–1355. 7696183

[31] Craven RJ, Cance WG, Liu ET (1995) The nuclear tyrosine kinase Rak associates with the retinoblastoma protein pRb. Cancer Res 55: 3969–3972. 7664264

[32] Brauer PM, Tyner AL (2009) RAKing in AKT: a tumor suppressor function for the intracellular tyrosine kinase FRK. Cell cycle 8: 2728–2732. 19652529 10.4161/cc.8.17.9389 PMC3005195

[33] Tanikawa C, Ueda K, Nakagawa H, Yoshida N, Nakamura Y, et al (2009) Regulation of protein Citrullination through p53/PADI4 network in DNA damage response. Cancer Res 69: 8761–8769. 19843866 10.1158/0008-5472.CAN-09-2280

[34] Chung KP, Chang YJ, Lai MS, Kuo RN, Cheng SH, et al (2010) Is quality of colorectal cancer care good enough? Core measures development and its application for comparing hospitals in Taiwan. BMC Health Serv Res 10: 27. 20105287 10.1186/1472-6963-10-27 PMC2835701

[35] Li P, Yao H, Zhang Z, Li M, Luo Y, et al (2008) Regulation of p53 target gene expression by peptidylarginine deiminase 4. Mol Cell Biol 28: 4745–4758. 18505818 10.1128/MCB.01747-07 PMC2493360

[36] Liu GY, Liao YF, Chang WH, Liu CC, Hsieh MC, et al (2006) Overexpression of peptidylarginine deiminase IV features in apoptosis of haematopoietic cells. Apoptosis 11: 183–196. 16502257 10.1007/s10495-006-3715-4

[37] Yu WP, Scott SA, Dong WF (2008) Induction of ID1 expression and apoptosis by the histone deacetylase inhibitor (trichostatin A) in human acute myeloid leukaemic cells. Cell proliferation 41: 86–97. 18211287 10.1111/j.1365-2184.2007.00499.x PMC6496488

[38] Healey MA, Deaton SL, Alder JK, Winnepenninckx V, Casero RA, et al (2010) Id1 overexpression is independent of repression and epigenetic silencing of tumor suppressor genes in melanoma. Epigenetics 5: 410–421. 20484992 10.4161/epi.5.5.11929 PMC3654680

[39] Rayess H, Wang MB, Srivatsan ES (2012) Cellular senescence and tumor suppressor gene p16. International journal of cancer Journal international du cancer 130: 1715–1725. 22025288 10.1002/ijc.27316 PMC3288293

[40] Li J, Xie L, Gan X, Liu B, Zhang Y, et al (2011) Association of inhibitor of differentiation 1 expression with human papillomaviruses infections in cervical carcinoma. International journal of gynecological cancer 21: 1276–1281. 21670698 10.1097/IGC.0b013e31821f7452

[41] Yasmeen A, Bismar TA, Kandouz M, Foulkes WD, Desprez PY, et al (2007) E6/E7 of HPV type 16 promotes cell invasion and metastasis of human breast cancer cells. Cell cycle 6: 2038–2042. 17721085 10.4161/cc.6.16.4555

[42] Zhang P, Zheng Y, Shi J, Zhang Y, Liu S, et al (2010) Targeting a novel N-terminal epitope of death receptor 5 triggers tumor cell death. J Biol Chem 285: 8953–8966. 20106985 10.1074/jbc.M109.070680 PMC2838317

[43] Wang W, Fang Y, Sima N, Li Y, Li W, et al (2011) Triggering of death receptor apoptotic signaling by human papillomavirus 16 E2 protein in cervical cancer cell lines is mediated by interaction with c-FLIP. Apoptosis 16: 55–66. 20882347 10.1007/s10495-010-0543-3

[44] Tseng CW, Monie A, Trimble C, Alvarez RD, Huh WK, et al (2008) Combination of treatment with death receptor 5-specific antibody with therapeutic HPV DNA vaccination generates enhanced therapeutic anti-tumor effects. Vaccine 26: 4314–4319. 18598733 10.1016/j.vaccine.2008.06.049 PMC2614388

[45] Kurita S, Higuchi H, Saito Y, Nakamoto N, Takaishi H, et al (2010) DNMT1 and DNMT3b silencing sensitizes human hepatoma cells to TRAIL-mediated apoptosis via up-regulation of TRAIL-R2/DR5 and caspase-8. Cancer Sci 101: 1431–1439. 20398055 10.1111/j.1349-7006.2010.01565.x PMC11158615

[46] Shivapurkar N, Toyooka S, Toyooka KO, Reddy J, Miyajima K, et al (2004) Aberrant methylation of trail decoy receptor genes is frequent in multiple tumor types. Int J Cancer 109: 786–792. 14999791 10.1002/ijc.20041

[47] Tan S, Hougardy BM, Meersma GJ, Schaap B, de Vries EG, et al (2012) Human papilloma virus 16 E6 RNA interference enhances cisplatin and death receptor-mediated apoptosis in human cervical carcinoma cells. Molecular pharmacology 81: 701–709. 22328720 10.1124/mol.111.076539

[48] Iliopoulos D, Oikonomou P, Messinis I, Tsezou A (2009) Correlation of promoter hypermethylation in hTERT, DAPK and MGMT genes with cervical oncogenesis progression. Oncol Rep 22: 199–204. 19513524 10.3892/or_00000425

[49] Narayan G, Arias-Pulido H, Koul S, Vargas H, Zhang FF, et al (2003) Frequent promoter methylation of CDH1, DAPK, RARB, and HIC1 genes in carcinoma of cervix uteri: its relationship to clinical outcome. Mol Cancer 2: 24. 12773202 10.1186/1476-4598-2-24 PMC156646

[50] Raveh T, Droguett G, Horwitz MS, DePinho RA, Kimchi A (2001) DAP kinase activates a p19ARF/p53-mediated apoptotic checkpoint to suppress oncogenic transformation. Nat Cell Biol 3: 1–7. 11146619 10.1038/35050500

[51] Cillo C, Cantile M, Faiella A, Boncinelli E (2001) Homeobox genes in normal and malignant cells. J Cell Physiol 188: 161–169. 11424082 10.1002/jcp.1115

[52] Strathdee G, Holyoake TL, Sim A, Parker A, Oscier DG, et al (2007) Inactivation of HOXA genes by hypermethylation in myeloid and lymphoid malignancy is frequent and associated with poor prognosis. Clin Cancer Res 13: 5048–5055. 17785556 10.1158/1078-0432.CCR-07-0919

[53] Kim DS, Kim MJ, Lee JY, Lee SM, Choi JY, et al (2009) Epigenetic inactivation of Homeobox A5 gene in nonsmall cell lung cancer and its relationship with clinicopathological features. Mol Carcinog 48: 1109–1115. 19554572 10.1002/mc.20561

[54] Raman V, Tamori A, Vali M, Zeller K, Korz D, et al (2000) HOXA5 regulates expression of the progesterone receptor. J Biol Chem 275: 26551–26555. 10875927 10.1074/jbc.C000324200

[55] Kathpalia VP, Mussak EN, Chow SS, Lam PH, Skelley N, et al (2006) Genome-wide transcriptional profiling in human squamous cell carcinoma of the skin identifies unique tumor-associated signatures. The Journal of dermatology 33: 309–318. 16700662 10.1111/j.1346-8138.2006.00075.x

[56] Illingworth RS, Gruenewald-Schneider U, Webb S, Kerr AR, James KD, et al.. (2010) Orphan CpG islands identify numerous conserved promoters in the mammalian genome. PLoS genetics 6. 10.1371/journal.pgen.1001134 PMC2944787 20885785

[57] Vogler M (2012) BCL2A1: the underdog in the BCL2 family. Cell death and differentiation 19: 67–74. 22075983 10.1038/cdd.2011.158 PMC3252829

[58] Pratt ZL, Zhang J, Sugden B (2012) The latent membrane protein 1 (LMP1) oncogene of Epstein-Barr virus can simultaneously induce and inhibit apoptosis in B cells. Journal of virology 86: 4380–4393. 22318153 10.1128/JVI.06966-11 PMC3318665

[59] Sharma PL, Chunduri H, Wise J, Mindley R, Rimland D (2012) Replication-independent expression of anti-apoptosis marker genes in human peripheral blood mononuclear cells infected with the wild-type HIV-1 and reverse transcriptase variants. Viral immunology 25: 12–20. 22239233 10.1089/vim.2011.0057 PMC3271366

[60] Nair PN, McArdle L, Cornell J, Cohn SL, Stallings RL (2007) High-resolution analysis of 3p deletion in neuroblastoma and differential methylation of the SEMA3B tumor suppressor gene. Cancer Genet Cytogenet 174: 100–110. 17452250 10.1016/j.cancergencyto.2006.11.017

[61] Lai HC, Lin YW, Chang CC, Wang HC, Chu TW, et al (2007) Hypermethylation of two consecutive tumor suppressor genes, BLU and RASSF1A, located at 3p21.3 in cervical neoplasias. Gynecologic oncology 104: 629–635. 17097722 10.1016/j.ygyno.2006.10.003

[62] Kuzmin I, Liu L, Dammann R, Geil L, Stanbridge EJ, et al (2003) Inactivation of RAS association domain family 1A gene in cervical carcinomas and the role of human papillomavirus infection. Cancer Res 63: 1888–1893. 12702579

[63] Mullink H, Jiwa NM, Walboomers JM, Horstman A, Vos W, et al (1991) Demonstration of changes in cytokeratin expression in condylomata accuminata in relation to the presence of human papilloma virus as shown by a combination of immunohistochemistry and in situ hybridization. Am J Dermatopathol 13: 530–537. 1725244 10.1097/00000372-199113060-00002

[64] McIntosh PB, Laskey P, Sullivan K, Davy C, Wang Q, et al (2010) E1–E4-mediated keratin phosphorylation and ubiquitylation: a mechanism for keratin depletion in HPV16-infected epithelium. Journal of cell science 123: 2810–2822. 20663917 10.1242/jcs.061978 PMC2915882

[65] Bryan JT, Brown DR (2000) Association of the human papillomavirus type 11 E1?E4 protein with cornified cell envelopes derived from infected genital epithelium. Virology 277: 262–269. 11080474 10.1006/viro.2000.0599

[66] Badaracco G, Venuti A, Sedati A, Marcante ML (2002) HPV16 and HPV18 in genital tumors: Significantly different levels of viral integration and correlation to tumor invasiveness. J Med Virol 67: 574–582. 12116007 10.1002/jmv.10141

[67] Raman V, Martensen SA, Reisman D, Evron E, Odenwald WF, et al (2000) Compromised HOXA5 function can limit p53 expression in human breast tumours. Nature 405: 974–978. 10879542 10.1038/35016125

[68] Rehman I, Cross SS, Catto JW, Leiblich A, Mukherjee A, et al (2005) Promoter hyper-methylation of calcium binding proteins S100A6 and S100A2 in human prostate cancer. The Prostate 65: 322–330. 16015609 10.1002/pros.20302

[69] Elder JT, Zhao X (2002) Evidence for local control of gene expression in the epidermal differentiation complex. Experimental dermatology 11: 406–412. 12366693 10.1034/j.1600-0625.2002.110503.x

[70] Fernandez-Fernandez MR, Rutherford TJ, Fersht AR (2008) Members of the S100 family bind p53 in two distinct ways. Protein Sci 17: 1663–1670. 18694925 10.1110/ps.035527.108 PMC2548378

[71] Quentmeier H, Eberth S, Romani J, Weich HA, Zaborski M, et al (2012) DNA methylation regulates expression of VEGF-R2 (KDR) and VEGF-R3 (FLT4). BMC cancer 12: 19. 22251800 10.1186/1471-2407-12-19 PMC3297533

[72] Kim J, Hwang J, Jeong H, Song HJ, Shin J, et al (2012) Promoter methylation status of VEGF receptor genes: a possible epigenetic biomarker to anticipate the efficacy of intracellular-acting VEGF-targeted drugs in cancer cells. Epigenetics 7: 191–200. 22395469 10.4161/epi.7.2.18973

[73] Philibert RA, Sears RA, Powers LS, Nash E, Bair T, et al (2012) Coordinated DNA methylation and gene expression changes in smoker alveolar macrophages: specific effects on VEGF receptor 1 expression. Journal of leukocyte biology 92: 621–631. 22427682 10.1189/jlb.1211632 PMC3427615

[74] Walker J, Smiley LC, Ingram D, Roman A (2011) Expression of human papillomavirus type 16 E7 is sufficient to significantly increase expression of angiogenic factors but is not sufficient to induce endothelial cell migration. Virology 410: 283–290. 21159359 10.1016/j.virol.2010.11.010 PMC3038585

[75] Hogart A, Nagarajan RP, Patzel KA, Yasui DH, Lasalle JM (2007) 15q11–13 GABAA receptor genes are normally biallelically expressed in brain yet are subject to epigenetic dysregulation in autism-spectrum disorders. Hum Mol Genet 16: 691–703. 17339270 10.1093/hmg/ddm014 PMC1934608

[76] Orellano EA, Rivera OJ, Chevres M, Chorna NE, Gonzalez FA (2010) Inhibition of neuronal cell death after retinoic acid-induced down-regulation of P2X7 nucleotide receptor expression. Mol Cell Biochem 337: 83–99. 19882109 10.1007/s11010-009-0288-x PMC4028074

[77] Farrell AW, Gadeock S, Pupovac A, Wang B, Jalilian I, et al (2010) P2X7 receptor activation induces cell death and CD23 shedding in human RPMI 8226 multiple myeloma cells. Biochim Biophys Acta 1800: 1173–1182. 20647033 10.1016/j.bbagen.2010.07.001

[78] Gorodeski GI (2009) P2X7-mediated chemoprevention of epithelial cancers. Expert Opin Ther Targets 13: 1313–1332. 19845494 10.1517/14728220903277249

[79] Schulze-Lohoff E, Hugo C, Rost S, Arnold S, Gruber A, et al (1998) Extracellular ATP causes apoptosis and necrosis of cultured mesangial cells via P2Z/P2X7 receptors. Am J Physiol 275: F962–971. 9843914 10.1152/ajprenal.1998.275.6.F962

[80] Leung KT, Chan KY, Ng PC, Lau TK, Chiu WM, et al (2011) The tetraspanin CD9 regulates migration, adhesion, and homing of human cord blood CD34+ hematopoietic stem and progenitor cells. Blood 117: 1840–1850. 21063023 10.1182/blood-2010-04-281329

[81] De Bruyne E, Bos TJ, Asosingh K, Vande Broek I, Menu E, et al (2008) Epigenetic silencing of the tetraspanin CD9 during disease progression in multiple myeloma cells and correlation with survival. Clin Cancer Res 14: 2918–2926. 18483358 10.1158/1078-0432.CCR-07-4489

[82] Zhong S, Fields CR, Su N, Pan YX, Robertson KD (2007) Pharmacologic inhibition of epigenetic modifications, coupled with gene expression profiling, reveals novel targets of aberrant DNA methylation and histone deacetylation in lung cancer. Oncogene 26: 2621–2634. 17043644 10.1038/sj.onc.1210041

[83] Gordon-Alonso M, Yanez-Mo M, Barreiro O, Alvarez S, Munoz-Fernandez MA, et al (2006) Tetraspanins CD9 and CD81 modulate HIV-1-induced membrane fusion. J Immunol 177: 5129–5137. 17015697 10.4049/jimmunol.177.8.5129

[84] Zhang S, Kodys K, Babcock GJ, Szabo G (2012) CD81/CD9 tetraspanins aid plasmacytoid dendritic cells in recognition of HCV-infected cells and induction of IFNalpha. Hepatology [Epub ahead of print]. 10.1002/hep.25827 PMC4511847 22577054

[85] Hernandez A (2005) Structure and function of the type 3 deiodinase gene. Thyroid 15: 865–874. 16131329 10.1089/thy.2005.15.865

[86] Martin-Subero JI, Ammerpohl O, Bibikova M, Wickham-Garcia E, Agirre X, et al (2009) A comprehensive microarray-based DNA methylation study of 367 hematological neoplasms. PLoS One 4: e6986. 19750229 10.1371/journal.pone.0006986 PMC2737286

[87] Huang MP, Rodgers KA, O’Mara R, Mehta M, Abuzahra HS, et al (2011) The thyroid hormone degrading type 3 deiodinase is the primary deiodinase active in murine epidermis. Thyroid 21: 1263–1268. 21936673 10.1089/thy.2011.0105

[88] Wong M, Fish EN (1998) RANTES and MIP-1alpha activate stats in T cells. J Biol Chem 273: 309–314. 9417081 10.1074/jbc.273.1.309

[89] Cocchi F, DeVico AL, Garzino-Demo A, Arya SK, Gallo RC, et al (1995) Identification of RANTES, MIP-1 alpha, and MIP-1 beta as the major HIV-suppressive factors produced by CD8+ T cells. Science 270: 1811–1815. 8525373 10.1126/science.270.5243.1811

[90] Cook DN, Beck MA, Coffman TM, Kirby SL, Sheridan JF, et al (1995) Requirement of MIP-1 alpha for an inflammatory response to viral infection. Science 269: 1583–1585. 7667639 10.1126/science.7667639

[91] Ohlschlager P, Quetting M, Alvarez G, Durst M, Gissmann L, et al (2009) Enhancement of immunogenicity of a therapeutic cervical cancer DNA-based vaccine by co-application of sequence-optimized genetic adjuvants. Int J Cancer 125: 189–198. 19358269 10.1002/ijc.24333

[92] Chen LM, Verity NJ, Chai KX (2009) Loss of prostasin (PRSS8) in human bladder transitional cell carcinoma cell lines is associated with epithelial-mesenchymal transition (EMT). BMC cancer 9: 377. 19849847 10.1186/1471-2407-9-377 PMC2770574

[93] Leyvraz C, Charles RP, Rubera I, Guitard M, Rotman S, et al (2005) The epidermal barrier function is dependent on the serine protease CAP1/Prss8. The Journal of cell biology 170: 487–496. 16061697 10.1083/jcb.200501038 PMC2171460

[94] Duenas-Gonzalez A, Lizano M, Candelaria M, Cetina L, Arce C, et al (2005) Epigenetics of cervical cancer. An overview and therapeutic perspectives. Mol Cancer 4: 38. 16248899 10.1186/1476-4598-4-38 PMC1291396

[95] Feng Q, Balasubramanian A, Hawes SE, Toure P, Sow PS, et al (2005) Detection of hypermethylated genes in women with and without cervical neoplasia. J Natl Cancer Inst 97: 273–282. 15713962 10.1093/jnci/dji041

[96] Flatley JE, McNeir K, Balasubramani L, Tidy J, Stuart EL, et al (2009) Folate status and aberrant DNA methylation are associated with HPV infection and cervical pathogenesis. Cancer Epidemiol Biomarkers Prev 18: 2782–2789. 19755648 10.1158/1055-9965.EPI-09-0493

[97] Yang N, Nijhuis ER, Volders HH, Eijsink JJ, Lendvai A, et al (2010) Gene promoter methylation patterns throughout the process of cervical carcinogenesis. Cell Oncol 32: 131–143. 20208141 10.3233/CLO-2009-0510 PMC4619050

[98] Kim JH, Choi YD, Lee JS, Lee JH, Nam JH, et al (2010) Assessment of DNA methylation for the detection of cervical neoplasia in liquid-based cytology specimens. Gynecol Oncol 116: 99–104. 19836067 10.1016/j.ygyno.2009.09.032

[99] Laurson J, Khan S, Chung R, Cross K, Raj K (2010) Epigenetic repression of E-cadherin by human papillomavirus 16 E7 protein. Carcinogenesis 31: 918–926. 20123756 10.1093/carcin/bgq027 PMC2864410

[100] Lin TS, Lee H, Chen RA, Ho ML, Lin CY, et al (2005) An association of DNMT3b protein expression with P16INK4a promoter hypermethylation in non-smoking female lung cancer with human papillomavirus infection. Cancer Lett 226: 77–84. 16004934 10.1016/j.canlet.2004.12.031

[101] Kraus I, Driesch C, Vinokurova S, Hovig E, Schneider A, et al (2008) The majority of viral-cellular fusion transcripts in cervical carcinomas cotranscribe cellular sequences of known or predicted genes. Cancer Res 68: 2514–2522. 18381461 10.1158/0008-5472.CAN-07-2776

[102] Matovina M, Sabol I, Grubisic G, Gasperov NM, Grce M (2009) Identification of human papillomavirus type 16 integration sites in high-grade precancerous cervical lesions. Gynecol Oncol 113: 120–127. 19157528 10.1016/j.ygyno.2008.12.004

[103] Peter M, Rosty C, Couturier J, Radvanyi F, Teshima H, et al (2006) MYC activation associated with the integration of HPV DNA at the MYC locus in genital tumors. Oncogene 25: 5985–5993. 16682952 10.1038/sj.onc.1209625

[104] Wentzensen N, Vinokurova S, von Knebel Doeberitz M (2004) Systematic review of genomic integration sites of human papillomavirus genomes in epithelial dysplasia and invasive cancer of the female lower genital tract. Cancer Res 64: 3878–3884. 15172997 10.1158/0008-5472.CAN-04-0009

[105] Yu T, Ferber MJ, Cheung TH, Chung TK, Wong YF, et al (2005) The role of viral integration in the development of cervical cancer. Cancer Genet Cytogenet 158: 27–34. 15771901 10.1016/j.cancergencyto.2004.08.021

