# Supplementary figures and images for: DNA Methylation Profiling across the Spectrum of HPV-Associated Anal Squamous Neoplasia
Source: PLoS One. 2012 Nov 30;7(11):e50533. doi: 10.1371/journal.pone.0050533 (PMC3511539; doi:10.1371/journal.pone.0050533)

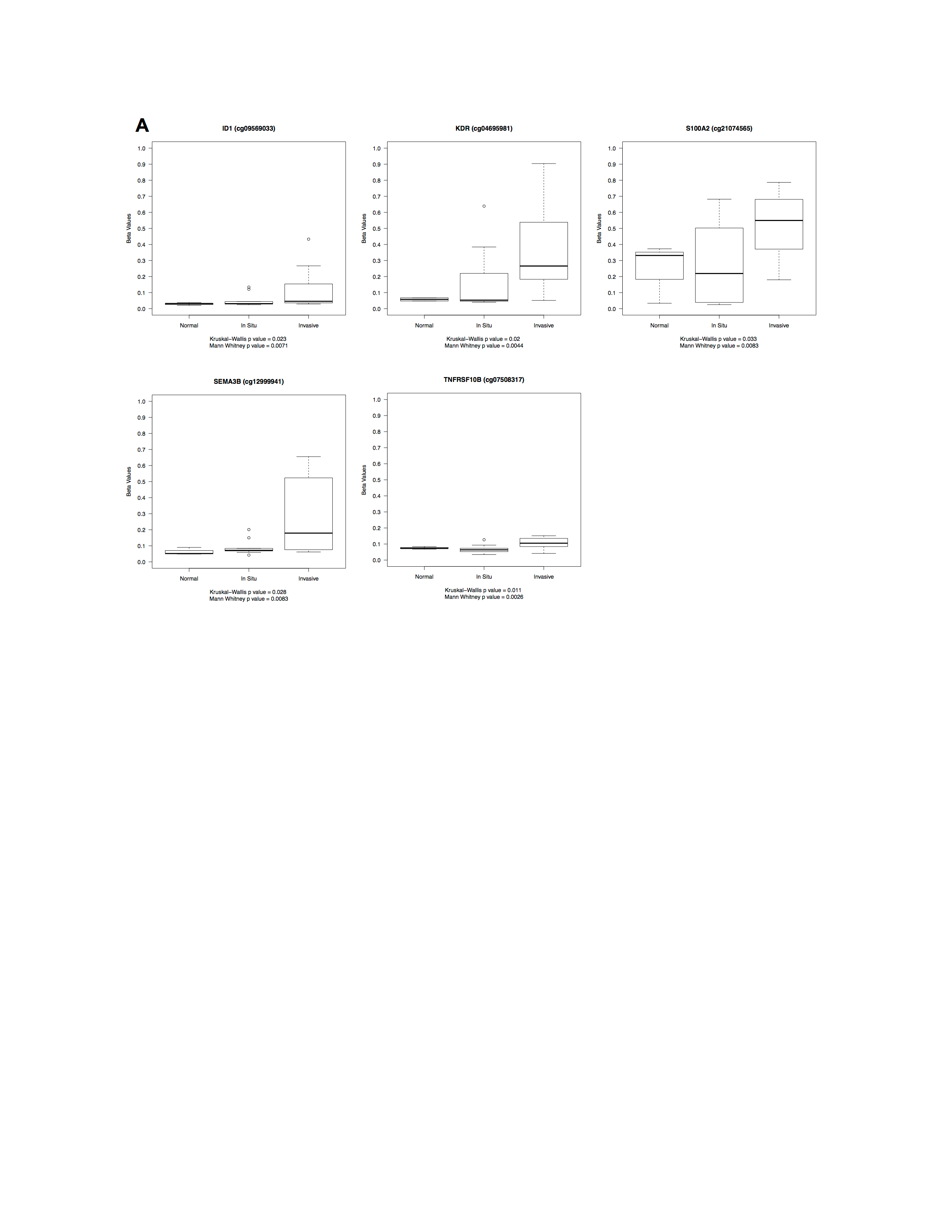

Supplement: Figure S1 — Boxplot Representations of All Differentially Methylated Loci. Boxplots illustrating methylation levels of 22 significant differentially methylated CpG sites representing 20 genes (Mann-Whitney p<0.01) across histologic subtypes (normal and SCC-IS vs. invasive CC). Three patterns are identified: A: Loci with low methylation levels (median beta value <0.3) across all tissues. B: Loci with high methylation levels (median beta value ≥0.3) across all tissues. The two CpG sites within the GABRA5 gene demonstrated reduced methylation with neoplastic progression C: Loci demonstrating little to no methylation in non-invasive tissues with significant levels of methylation in invasive SCC. (TIF) [file pone.0050533.s001.tif]
