# Supplementary material for: DNA Methylation Profiling across the Spectrum of HPV-Associated Anal Squamous Neoplasia
Source: PLoS One. 2012 Nov 30;7(11):e50533. doi: 10.1371/journal.pone.0050533 (PMC3511539; doi:10.1371/journal.pone.0050533)
Supplement: Table S1 — Genes with Differentially Methylated CpG Loci in the Progression of Anal Neoplasia with Full Annotation. (DOC) [file pone.0050533.s002.doc]

**Table S1.** Genes with Differentially Methylated CpG Loci in the Progression of Anal Neoplasia with Full Annotation

| **Symbol** | **Methylation and Tumorigenesis** | **Interaction with HPV** | **Accession** | **Synonym** | | **Distance to TSS** | **CpG Island** | |
| --- | --- | --- | --- | --- | --- | --- | --- | --- |
| **Growth Regulation and Cell Cycle Control** | | | | | | | | |
| ***TGFB3*** | TSG; Methylation associated with promoter 2 alters expression . | E6 and E7 decrease expression of TGFβ3 family member, TGFβ2 . E7 represses TGFβ2 promoter by releasing E2F from pRb . | NM_003239.1 | FLJ16571, TGF-beta3 | | 58 | N | |
| ***FRK*** | TSG; Loss of growth inhibition ; No reports of methylation in cancer. | Phosphorylates PTEN; Interacts with Rb . | NM_002031.2 | GTK, RAK, PTK5 | | -258 | N | |
| ***PADI4*** | Regulates histone methylation; Transcriptional repressor of p53 or estrogen regulated genes . | p53 response to DNA damage/cell-stress. Recruited by p53 to genes to repress transcription (e.g. p21) . | NM_012387.1 | PAD, PDI4, PDI5, PADI5 | | -1158 | N | |
| ***ID1*** | Transcriptional repressor of TSG, including TSP1, CDKN2A (p16) and CDKN1A (p21), which are frequently epigenetically silenced in cancer . Low expression reported due to aberrantly deacetylated histones with little methylation. | Inhibits the activity of ETS transcription factors decreased the transcription of p16. Elevated protein in HPV16+ cervical cancers compared to other HPV types. In breast cancer cells, transfection with E7/E7 upregulated ID1, increasing invasiveness. | NM_002165.2 | ID | | -659 | Y | |
| **Differentiation** | | | | | | | | |
| ***S100A2*** | TSG; Hypermethylated in several tumors. Unmethylated in normal keratinocytes during differentiation . | No association with HPV oncogenes reported; Binds and may modulate p53 . | NM_005978.3 | CAN19, S100L, MGC111539 | | 36; -1186 | N; N | |
| ***KRT1*** | Involved in tumor cell differentiation; Expression changes in anal condyloma . | HPV E1^E4 protein disrupts keratin which might compromise epithelial integrity, thus facilitating virion release . | NM_006121.2 | K1, CK1, EHK1, KRT1A | | -798 | N | |
| ***KRT5*** | Involved in tumor cell differentiation; Expression changes in anal condyloma . | HPV E1^E4 protein disrupts keratin which might compromise epithelial integrity, thus facilitating virion release . | NM_000424.2 | K5, CK5, DDD, EBS2, KRT5A | | 196 | Y | |
| **Angiogenesis** | | | | | | | | |
| ***FLT1*** | Anti-angiogenic; Epigenetic silencing observed in several tumor sites (prostate, lymphoma). | HPV oncogenes upregulate VEGF expression and increases angiogenesis. | NM_002019.2 | FLT, VEGFR1 | | -302 | Y | |
| ***KDR*** | Pro-angiogenic; Epigenetic silencing observed in several tumor sites (prostate, lymphoma) .. | HPV oncogenes upregulate VEGF expression and increases angiogenesis. | NM_002253.1 | FLK1, CD309, VEGFR, VEGFR2 | | -445 | Y | |
| **Apoptosis** | | | | | | | | |
| ***DAPK1*** | Epigenetic silencing in HPV-associated anal and cervical cancer. | Counteracts oncogene-induced transformation by activation of a p53-dependent apoptotic checkpoint. | NM_004938.1 | DAPK, DKFZp781I035 | | -10 | Y | |
| ***HOXA5*** | Expression strongly correlated with promoter methylation. Loss of HOXA5 found in tumors. | Upregulates p53 expression, an HPV target . Loss of HOXA5 may be an additional mechanism to reduce p53 activity. | NM_019102.2 | HOX1, HOX1C, HOX1.3, MGC9376 | | -479 | Y | |
| ***TNFRSF10B*** | Knockdown of DNMTs resulted in upregulation of TNFRSF10B expression. Methylation of TNFRSF family members (10C and 10D) reported in cervical cancer . | Cell surface expression not related to E6 expression level. Possible target to increase sensitivity to apoptosis. | NM_147187.1 | DR5, CD262, KILLER, TRICK2/B/2A/2B, ZTNFR9, TRAILR2, KILLER/DR5 | | 198 | Y | |
| ***BCL2A1*** | Upregulated in tumors. Reported orphan CpG Islands, unclear if methylated or of relevance to cancer. | Direct transcription target of NF-kappa B in response to inflammatory mediators . No reported interaction with HPV, however upregulated by HIV and EBV. | NM_004049.2 | GRS, BFL1, HBPA1, BCL2L5 | | -1127 | Y | |
| ***SEMA3B*** | TSG; Hypermethylated in several cancers. Chromosomal region 3p21.3 contains several hypermethylated genes in cervical cancer, including BLU and RASSF1a . | Higher methylation of BLU in HPV-positive cervical tumors. | NM_004636.2 | SemA, SEMA5, SEMAA, semaV, LUCA-1, FLJ34863 | | -110 | N | |
| **Other Processes** | | | | | | | | |
| ***GABRA5*** | Part of an imprinted locus at 15q11-13. Loss may contribute to multiple neuro-developmental disorders such as Prader-Willi syndrome, Angelman syndrome and autism.. No association with cancer reported. | No association with known HPV-associated pathways or malignancies reported. | NM_000810.2 | . | -1016; -862 | | | N; N |
| ***P2RX7*** | Ionotropic ATP receptor that mediates cell death. Activation induces cell death in multiple myeloma . No reports on methylation. | P2RX7 is an important pro-apoptotic modulator in skin epithelium. P2X7 agonists have been proposed as a chemopreventive strategy in skin neoplasia. P2RX7 agonist treatment of rat mesangial cells results in an increase in protein levels of p53 with increase in apoptosis. | NM_177427.2 | P2X7, MGC20089 | -597 | | | N |
| ***CD9*** | Regulates cell migration, adhesion of hematopoietic stem cells. Epigenetic silencing associated with disease progression in multiple myeloma and non-small cell lung tumors. | Loss of CD9 expression results in host cell membrane alterations facilitating HIV viral entry. CD9 is required for recognition of Hepatitis C virus and induction of host dendritic cell response. | NM_001769.2 | 5H9, BA2, P24, GIG2, MIC3, MRP-1, BTCC-1, DRAP-27, TSPAN29 | -585 | | | Y |
| ***DIO3*** | Paternally imprinted. Significantly hypermethylated in B-cell, T-cell and myeloid malignancies. Reduced levels via altered methylation could results in altered expression of thyroid hormone responsive genes. | DIO3 is the primary thyroid deiodinase in murine epidermis. DIO3 is expressed in keratinocytes and appears to prevent skin from becoming hyperproliferative. | NM_001362.2 | D3, 5DIII, TXDI3, DIOIII | 230 | | | Y |
| ***CCL3*** | Monokine with inflammatory and chemokinetic properties; Binds to CCR1, CCR4 and CCR5; thus loss would reduce immune response. No data on methylation. | One of the major HIV-suppressive factors produced by CD8+ T-cells.; Important mediator of virus (coxsackie and influenza)-clearance and inflammatory response. CCL3 administered as an adjunct to an HPV-16 E7-based DNA vaccine against cervical cancer results in an enhanced tumor response. | NM_002983.1 | MIP1A, SCYA3, G0S19-1, LD78ALPHA, MIP-1-alpha | -543 | | | N |
| ***PRSS8*** | Promoter DNA hypermethylation shown in various cancer cell lines . Important for epidermal differentiation and integrity of permeability barrier. | No association with known HPV-associated pathways or malignancies. | NM_002773.2 | CAP1, PROSTASIN | 134 | | | Y |
